# Insulin Resistance and Blood-Brain Barrier Dysfunction Underlie Neuroprogression in Bipolar Disorder

**DOI:** 10.3389/fpsyt.2021.636174

**Published:** 2021-05-25

**Authors:** Cynthia Calkin, Christie McClelland, Kathleen Cairns, Lyna Kamintsky, Alon Friedman

**Affiliations:** ^1^Department of Psychiatry, Dalhousie University, Halifax, NS, Canada; ^2^Department of Medical Neuroscience, Dalhousie University, Halifax, NS, Canada; ^3^Nova Scotia Health, Halifax, NS, Canada; ^4^Departments of Cell Biology and Physiology, Ben-Gurion University of the Negev, Beer-Sheva, Israel

**Keywords:** bipolar disorder, blood-brain barrier, insulin resistance, neuroprogression, vascular damage, inflammation

## Abstract

Bipolar disorder (BD) often progresses to a more chronic and treatment resistant (neuroprogressive) course. Identifying which patients are at risk could allow for early intervention and prevention. Bipolar disorder is highly comorbid with metabolic disorders including type II diabetes mellitus (T2DM), hypertension, obesity, and dyslipidemia. Our studies have shown that insulin resistance (IR) is present in over 50% of patients with BD and that IR might underlie the progression of BD. While no confirmed predictors exist for identifying which patients with BD are likely to develop a more chronic course, emerging evidence including our own studies suggest that IR and related inflammatory pathways lead to impairments in blood-brain barrier (BBB) functioning. For the first time in living psychiatric patients, we have shown that the severity of BBB leakage is proportional to BD severity and is associated with IR. In this hypothesis paper we (i) highlight the evidence for a key role of IR in BD, (ii) show how IR in BD relates to shared inflammatory pathways, and (iii) hypothesize that these modulations result in BBB leakage and worse outcomes in BD. We further hypothesize that (iv) reversing IR through lifestyle changes or the actions of insulin sensitizing medications such as metformin, or optimizing BBB function using vascular protective drugs, such as losartan, could provide novel strategies for the prevention or treatment of neuroprogressive BD.

## Introduction

**Figure 1:**
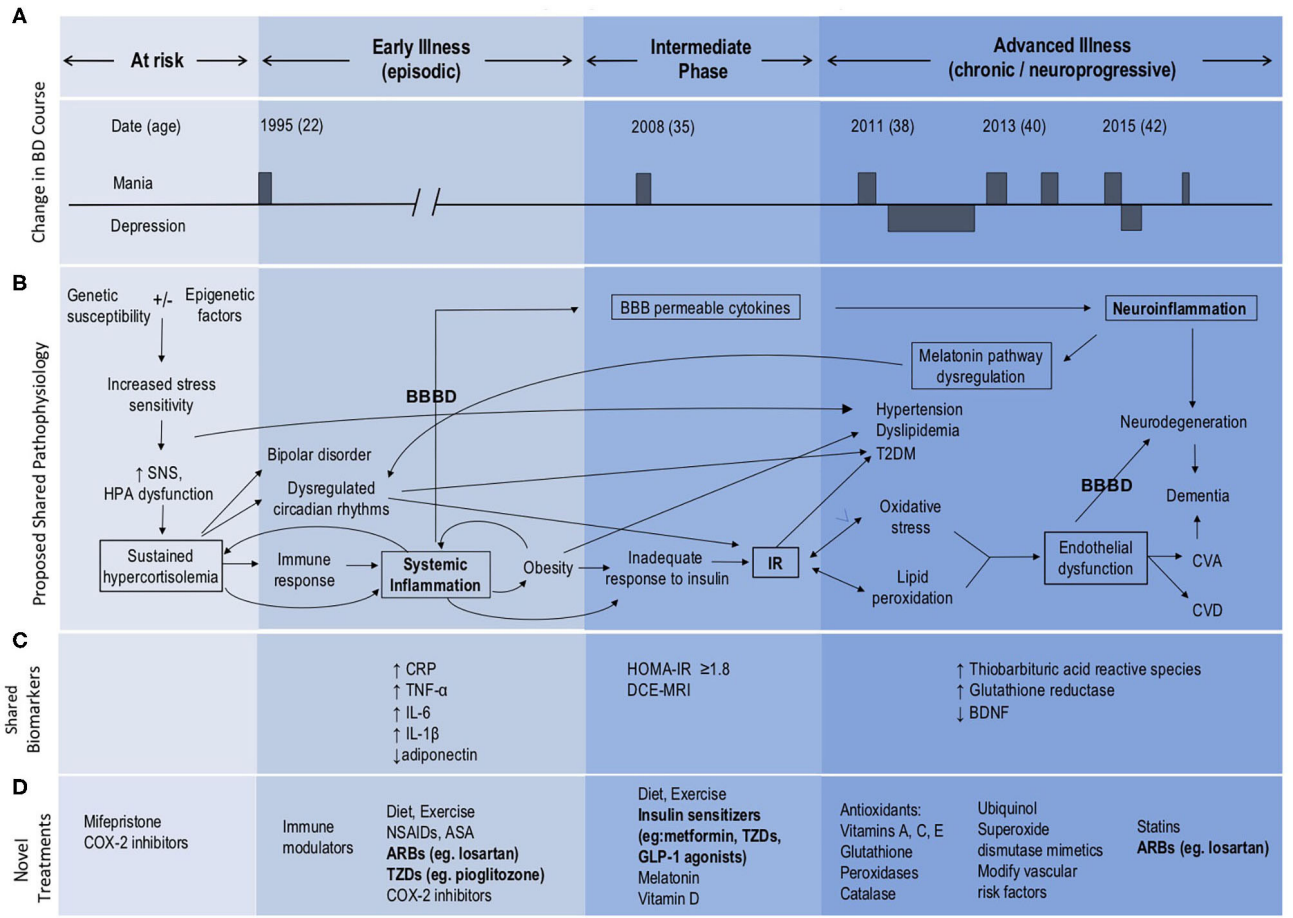
Model of BD neuroprogression mediated by IR and BBBD. ARBs, Angiotensin II receptor blockers; ASA, acetylsalicylic acid; BBB, blood-brain barrier; BBBD, blood-brain barrier dysfunction; BDNF, brain-derived neurotrophic factor; CNS, central nervous system; COX-2 inhibitors, Cyclooxygenase-2 inhibitors; CRP, C-reactive protein; CVA, cerebrovascular accident; CVD, cardiovascular disease; GLP, glucagon-like peptide; HOMA-IR, Homeostatic Model Assessment—Insulin Resistance; HPA, hypothalamic pituitary adrenal; IL, interleukin; IR, insulin resistance; NSAIDs, Non-steroidal anti-inflammatory drugs; SNS, sympathetic nervous system; TNF, tumor necrosis factor; T2DM, type 2 diabetes mellitus; TZDs, thiazolidinediones. This figure has been modified from the original published figure by Calkin C V., Insulin resistance takes center stage: a new paradigm in the progression of bipolar disorder, published in Annals of Medicine. Taylor and Francis Ltd; 2019.

Bipolar disorder is a mood disorder affecting up to 5% of the population, leading to significant morbidity and premature mortality. Mood dysregulation occurs in conjunction with symptoms affecting sleep, energy, interests/motivation, appetite/weight, concentration, speech, thought process, and judgment. Patients experience episodes of mania with or without depressive episodes in bipolar I disorder and recurrent episodes of hypomania and depression in bipolar II disorder. Treatment-resistant disease progression (neuroprogression) is not uncommon in bipolar patients and includes a shift toward more severe, prolonged and frequent mood episodes, including rapid cycling (a minimum of 4 discrete mood episodes yearly) (Figure 1A), and poor functional outcomes (1, 2). Until now there have been no validated predictors for which patients will progress to this advanced course and there are no corresponding treatments. Increasing evidence suggests that comorbid metabolic dysregulation, and specifically IR might underlie the progression of BD (3). We have found that those with comorbid T2DM or IR were more likely to develop a chronic course of BD, more rapid cycling, and were less likely to respond to lithium compared to those without metabolic dysregulation (4). Similarly, poor outcomes in BD with comorbid IR have been reported, including worse cognitive decline (5), memory impairment (6), and poor response to mood stabilizers in general (7). Understanding mechanisms underlying these findings could lead to novel therapeutic or adjunctive treatment strategies.

Insulin resistance is an inflammatory state which affects the vasculature and can lead to endothelial changes in the BBB (8) (Figure 1B). We have recently examined the potential role of BBB dysfunction (BBBD) in BD using dynamic contrast-enhanced magnetic resonance imaging (DCE-MRI). We found that BD patients with extensive BBB leakage had a more chronic course, greater severity of depression and anxiety, and poorer overall functioning compared with BD patients with normal BBB permeability (9). Further, we found that all patients with extensive BBB leakage also had IR. Expanding on our earlier hypothesis that IR plays a role in the development of neuroprogression in BD (3), we now propose that *both* IR and BBBD are biomarkers for neuroprogression and advancement of the illness process. We suggest that neuroprogression in patients with BD may be the *result* of comorbid IR and its effect on the integrity of the BBB, as mediated through shared inflammatory pathways (Figure 1B).

## Metabolic Dysregulation In BD

Patients with BD are symptomatic almost half of their lives (10). The leading cause of death in BD is cardiovascular disease with a 2.3-fold increased risk in patients with BD compared to the general population (11). Rates of cardiovascular risk factors including obesity, hypertension, dyslipidemia, metabolic syndrome and T2DM are all higher in patients with BD (12–14), and bipolar patients with comorbid T2DM have higher rates of hypertension, obesity, and a more chronic course (15). Obesity is a chronic, proinflammatory state, and adipose tissue secretes cytokines and inflammatory mediators (16) (Figures 1B,C) leading to further subsequent systemic inflammation and worsening obesity. This eventually results in an inadequate increase in insulin in response to plasma glucose, leading to IR with progression to glucose intolerance and eventually T2DM (17). We have reported that BD patients with comorbid obesity also have a more chronic course of illness and poor response to lithium (18). Similarly, worse outcomes in BD have been reported with comorbid IR, including worse cognitive decline (5).

Insulin resistance is present in more than half of all bipolar patients and is associated with a three times higher likelihood of a chronic course of illness with significantly more mood episodes including rapid cycling compared to those without metabolic dysregulation (4). These findings remained significant after controlling for the potential effects of age, body mass index (BMI), and lifetime exposure to antipsychotic medication (4). This is important, given that many medications used to treat BD can also lead to the development of metabolic dysfunction (19), although the association between BD and metabolic dysregulation was described as early as 1921, well before the advent of psychotropic medications (20, 21). We have also reported that psychiatric morbidity in BD increases 12-fold following the onset of IR, and we have identified IR as an early manifestation or possible predictor of progression of BD (22).

Insulin resistance might also be useful in predicting poor response to treatment. In a 2015 study, we found that bipolar patients with either T2DM or IR were 8.4 times more likely to have a poor response to lithium (the gold-standard treatment) than bipolar patients with normal glucose metabolism (4). An inverse relationship between IR and response to lithium was demonstrated, such that as IR progressed, a poorer response to lithium was found (4). Similarly, Steardo et al. (7) found that bipolar patients with comorbid IR/T2DM were 4.3 times more likely to fail treatment with *any* mood stabilizer, including lithium. Furthermore, we found that IR *preceded* neuroprogression of BD (22), highlighting the potential to modify BD trajectory through early treatment of IR. Together, these findings stress the importance of monitoring IR in BD patients (23, 24) for early identification of neuroprogression and targeted treatment strategies. Targeting IR in bipolar patients may not only facilitate BD remission; but could also decrease the risk for T2DM, cardiovascular disease and dementia (3).

## Hypothalamic Pituitary Adrenal (HPA) Dysfunction Leads To Systemic Inflammation

We identify the key role of the HPA axis in the development of both BD and IR (25–27), via induction of sustained hypercortisolemia (Figure 1B). Sustained hypercortisolemia causes the body to mount an immune response (25, 28), leading to systemic inflammation (29, 30), obesity, and increased risk of IR. Bipolar disorder is also associated with abnormalities of HPA axis activity, including increased levels of cortisol and adrenocorticotropic hormone (ACTH) (26, 27) along with disruption in the normal diurnal variation of cortisol. Specifically there is an absence of expected cortisol troughs at night (31) and higher than usual daytime elevations (32), contributing to circadian rhythm dysfunction. Bipolar patients also have altered sleep and cortisol levels even when euthymic (33) and poor sleep initiation and frequent nighttime awakenings increase risk for IR/T2DM (34).

## Systemic Inflammation Leads To Neuroinflammation And Neuroprogression

We further propose that systemic inflammation and IR increase the risk of endothelial injury, dysfunction of the BBB and subsequent neuroinflammation (8) (Figure 1B). Neuroinflammation then further amplifies BBBD, creating a self-reinforcing positive feedback loop that exacerbates BD, and contributes to its progression. In addition, hyperglycemia and hyperinsulinemia activate the renin-angiotensin system (RAS) which contributes to the development of hypertension and endothelial dysfunction Endothelial dysfunction results in microvascular and macrovascular changes, which when occurring within the brain lead to impairments in BBB functions. Given that even mild hyperglycemia can lead to profound dysfunction of the BBB (8), it is possible that early intervention with lifestyle and dietary changes (35) at the IR stage prior to the development of hyperglycemia could prevent or mitigate these effects on the BBB and development of neuroprogression. Further, targeting factors such as hypercortisolemia, sleep disturbances, IR, BBBD and/or neuroinflammation may offer novel therapeutic avenues for the management of BD and in preventing neuroprogression (Figure 1D).

## Systemic Inflammation In BD And IR

Mood disorders are understood to develop from a combination of genetic and environmental factors, which ultimately lead to a broad spectrum of clinical presentations. While the pathophysiology of these processes remains largely elusive, increasing evidence supports a key role of inflammatory cascades in BD (36, 37). Inflammation is defined as a non-specific state, known to be caused by both internal and external factors and may represent the body's response as a defense to a perceived threat (25, 37). Patients with systemic autoimmune diseases have an increased propensity for the development of BD, and several increased peripheral proinflammatory mediators have been reported in BD (38). Further, markers of neuroinflammation are present in the cerebrospinal fluid of living patients with BD (39), and post-mortem studies have demonstrated increased inflammatory markers in the frontal cortex (40). Along with BD, it is generally accepted that a mild inflammatory state occurs in major depressive disorder (MDD) and schizophrenia (41). Long-term exposure to cytokines can lead to depressive episodes in euthymic patients receiving immune therapy with INF-alpha (42–44). Similarly, the administration of pro-inflammatory cytokines may lead to depressive symptoms in healthy controls (45–47). Blockade of TNF-type cytokines in depressed subjects with comorbid diseases including rheumatoid arthritis, psoriasis, and cancer was found to significantly *reduce* depressive symptoms (48, 49). Additionally, improvement in psychiatric symptoms has been demonstrated in schizophrenia patients treated with anti-inflammatory drugs for other indications (50).

The most prominent cytokines associated with mood disorders include: interleukin-1 beta (IL-1ß), interleukin-6 (IL-6), tumor necrosis factor alpha (TNF-alpha), and C-reactive protein (CRP) (51) (see Figure 1C). Interleukin-6 was shown to be increased in BD patients when unwell. Interestingly, levels of IL-6 were decreased following 6 weeks of mood stabilizing treatment (52). Indeed, increased activity of pro-inflammatory cytokines and an imbalance with anti-inflammatory cytokines have been implicated in the development of BD and neuroprogression (51–53) and (see Figure 1C). Of particular interest, varying cytokine profiles have been identified in distinct mood states of BD, including in mania, depression, euthymia and in healthy controls (54, 55). Specifically, manic episodes are associated with a prominent pro-inflammatory profile state (38). It has also been shown that inflammatory markers positively correlate with symptom severity in BD (29, 56, 57). In late-stage BD more extreme elevations in these serum markers have been found, especially TNF-alpha. As underscored by Benedetti et al. (58), increased inflammation has been linked to other known hallmarks of BD, including white matter changes and structural alterations in the prefrontal cortex, hippocampus, and amygdala (59, 60).

Practically, inflammatory profiles could prove to be useful in predicting treatment response to antidepressants in depressed states. The presence of neuroinflammation has been shown to result in a decreased response to some antidepressants (61). Specifically, in a systematic review, the presence of an inflammatory state (raised serum CRP and IL-6) in MDD patients correlated with poor outcome and poor response to predominantly serotonergic antidepressants (61). A better response to antidepressant regimes was demonstrated with add-on noradrenergic, dopaminergic, or glutamatergic action (61). In rats with a depression-like phenotype, augmentation with acetylsalicylic acid (ASA), a non-selective cyclo-oxygenase (COX) inhibitor and anti-inflammatory, enhanced the efficacy of fluoxetine (62). Levels of immunological markers could also help to predict the efficacy of some medications in BD, such as lithium response (29, 63–65). Overall evidence suggests that an activated inflammatory response is associated with treatment resistance in general and is possibly indicative of a different (i.e., neuroprogressive) disease phase. This draws a similarity to our findings of treatment resistance in bipolar patients with comorbid IR and thus, possibly, indicates a shared inflammatory mechanism.

Over years of research, various signaling pathways have been associated with mood disorders, including alterations in 5-hydroxytryptamine (5-HT) receptor functioning, neurotrophins, and the HPA axis (25, 28, 30). Immune dysregulation has also become a focused area of this research. However, the general framework regarded immune alterations as an association or *consequence* of mood disorders, as opposed to a causative factor. A growing body of research has questioned this framework. Indeed, genetic studies have shown that immune alterations are detectable even before the onset of BD (58). Moreover, inflammatory serum markers are also elevated in adolescents with BD prior to the development of a clear illness course (66). A recent systematic review by Mucci et al. (51) highlights inflammatory processes as the mechanism contributing to the onset of mood disorders following a stressful stimuli.

It has also been found that an increased production of cortisol in Cushing's disease leads to depressive and manic symptoms and neurocognitive deficits, and affects various central nervous system (CNS) regions (67). In addition to other commonalities, increased oxidative stress has been identified in both BD and T2DM. Insulin resistance initially stimulates an increase in metabolic activity, resulting in elevated production of reactive oxygen species (ROS), and a subsequent increase in inflammatory cytokines, such as TNF-alpha, Interleukin Beta (IL-ß), and monocyte chemoattractant protein-1 (MCP-1) (68). Importantly, oxidative stress markers such as nitric oxide (NO) and ROS have also been suggested to play a role in the pathophysiology of BD (57). Since oxidative stress and inflammation may contribute to both BD and IR, IR-related inflammation could underlie and precede BD neuroprogression, triggering a transition into a more chronic and treatment-resistant course of illness (3, 69, 70) (see Figures 1A,B). Utilizing a growing body of research, we further propose that alterations in BBB functions play a critical role in this pathogenic cascade. Specifically, we suggest that: (i) IR and associated inflammation underlie microvascular injury and BBB dysfunction; and (ii) that high BBB permeability to serum components facilitates further neuroinflammation and dysfunction of the neurovascular unit—including neuronal networks, that underlie the chronic and neuroprogressive course of BD.

## Blood-Brain Barrier Dysfunction In BD

The BBB is a complex structural and functional interface tightly regulating molecular exchange between the circulation and brain tissue. The BBB is formed by endothelial cells, linked together by tight junction proteins, and surrounded by pericytes, and astroglial foot processes (71, 72). This complex structure restricts harmful molecules in the blood from entering the brain, while facilitating the entry of essential nutrients and removal of waste products (73, 74). The BBB is integral to healthy brain functioning, and pathological BBB has been associated with autoimmune diseases (75) and common neurological conditions (76–79). Increase in BBB permeability can be inferred indirectly, by measuring whether a patient's CSF has a high concentration of molecules that are normally excluded from the brain, for example, albumin or urate (80). High levels of these BBBD markers have been associated with MDD in elderly women, as well as suicidality in unipolar MDD patients (81–83)—supporting a possible role for BBBD in mood disorders. Post-mortem evidence of BBBD has been demonstrated in neuropsychiatric disorders such as dementia (84) and depression (85). Recent advances in MRI techniques have allowed direct assessment of BBB functionality in living patients, demonstrating BBBD in pathological conditions such as traumatic and ischemic injuries (86, 87), epilepsy (77), dementia (78) multiple sclerosis (79), and systemic lupus erythematosus (SLE) (75).

The understanding of the molecular mechanisms involved in BBBD had been largely derived from experimental animal models. This growing body of evidence suggests that: (i) systemic inflammation is associated with injury to brain (and other organs) microvasculature, resulting in BBBD (88), and (ii) BBBD allows leakage of serum macromolecules into the brain, resulting in glial activation, neuro-inflammation, network reorganization and delayed neurodegeneration as well as further BBBD (71, 73, 89).

One widely studied example of a serum macromolecule that leaks into the brain following BBB dysfunction is albumin (80)—the most abundant protein in the blood. Once leaking into the brain, albumin has been shown to trigger glial transformation, by activation of the pro-inflammatory transforming growth factor beta (TGF-ß) pathway (80, 89, 90), leading to cytokine secretion, synaptogenesis and neurodegeneration (77, 87, 88). In mouse models of social defeat, stress was also linked to increased BBB permeability and subsequent leakage of molecules into the brain (91). Specifically, the tight junction protein claudin-5 was downregulated and promoted peripheral IL-6 passage across the BBB (91). Claudin-5 expression was similarly downregulated in depressed patients (92). Using immunohistochemistry and qRT-PCR, (92) a key role for claudin-5 which was reduced in the hippocampus in post-mortem human brain tissues in people diagnosed with MDD or schizophrenia was also demonstrated. Interestingly, levels of claudins including expression of claudin-5 correlated with disease duration and age of psychiatric disorder in these post-mortem studies (92). Indeed, BBB associated tight junction disruption could be a major step in the development of various psychiatric pathologies. This is further supported by post-mortem analysis of patients with MDD, showing reduced astrocytic coverage of the BBB in the orbitofrontal cortex (85). Notably, post-mortem studies in human patients with BD have also demonstrated neuroinflammatory changes, microglial activation, and oligodendrocyte dysfunction (93). To add to the animal and post-mortem studies linking BD, neuroinflammation, and BBBD, our group has recently conducted the first ever BBB imaging study in living bipolar patients (9). Using DCE-MRI, we have demonstrated that extensive BBB leakage affects 28% of BD patients (9), and that these patients had greater psychiatric morbidity, compared to BD patients with normal BBB function. Extensive BBB leakage was found to be associated with worse depression, anxiety and socio/occupational dysfunction, chronic illness course with more frequent and/or severe manic/depressive episodes (9). Importantly, all bipolar patients with extensive BBB leakage also had IR, supporting the hypothesis that IR-related inflammation may contribute to BBBD and BD progression. Our results suggest BBBD could indeed be a mechanistic link between systemic inflammation, IR and BD neuroprogression. Moreover, repair of the BBB may, thus, prove to be a novel and effective approach for maintaining brain health and facilitating BD remission.

## IR And Blood-Brain Barrier Dysfunction

Our finding that all BD patients with extensive BBB leakage also had IR (9), highlights a potential causal link between IR and BBBD. While animal and human studies suggest that inflammation is the likely mechanism connecting IR and BBBD, the direct mechanistic link between IR and BBBD in BD patients has yet to be studied. Evidence for the role of IR in BBBD can be found in animal studies of hyperglycemia, showing that hyperglycemia causes inflammation and damage to the BBB (8). Notably, numerous overlapping pathways are involved in the inflammatory states of both IR and BBBD, including vascular endothelial growth factor (VEGF) and protein kinase C (PKC) (94). Further evidence supporting a possible link between BBBD and IR comes from studies of BBBD in T2DM. Impairment of the BBB is now accepted as one of the key mechanisms leading to diabetic encephalopathy (95). The structural integrity and transport function of the BBB is compromised in T2DM, through pathways of oxidative stress and chronic inflammation (95). BBBD in T2DM has also been suggested to play a role in neuronal dysfunction (96), and cognitive impairment (95). Further, both human and animal studies have demonstrated a role for insulin in synaptic viability, dendritic spine formation, cerebral bioenergetics and suggest that insulin dysregulation can lead to diseases of pathological aging (97). Specifically, a relationship between IR in the periphery and the brain in Alzheimer's disease suggests that peripheral IR might precede accumulation of amyloid ß protein in Alzheimer's (97). Applying this concept to the current body of evidence in IR and BD, we postulate that BBBD is likely to be a key mechanism contributing to BD neuroprogression, as *mediated by* IR and shared inflammatory pathways (Figure 1B).

## Intracellular Mechanisms Supporting A Link Between IR, Inflammation, BBBD, And Neuroprogression

**Figure 2:**
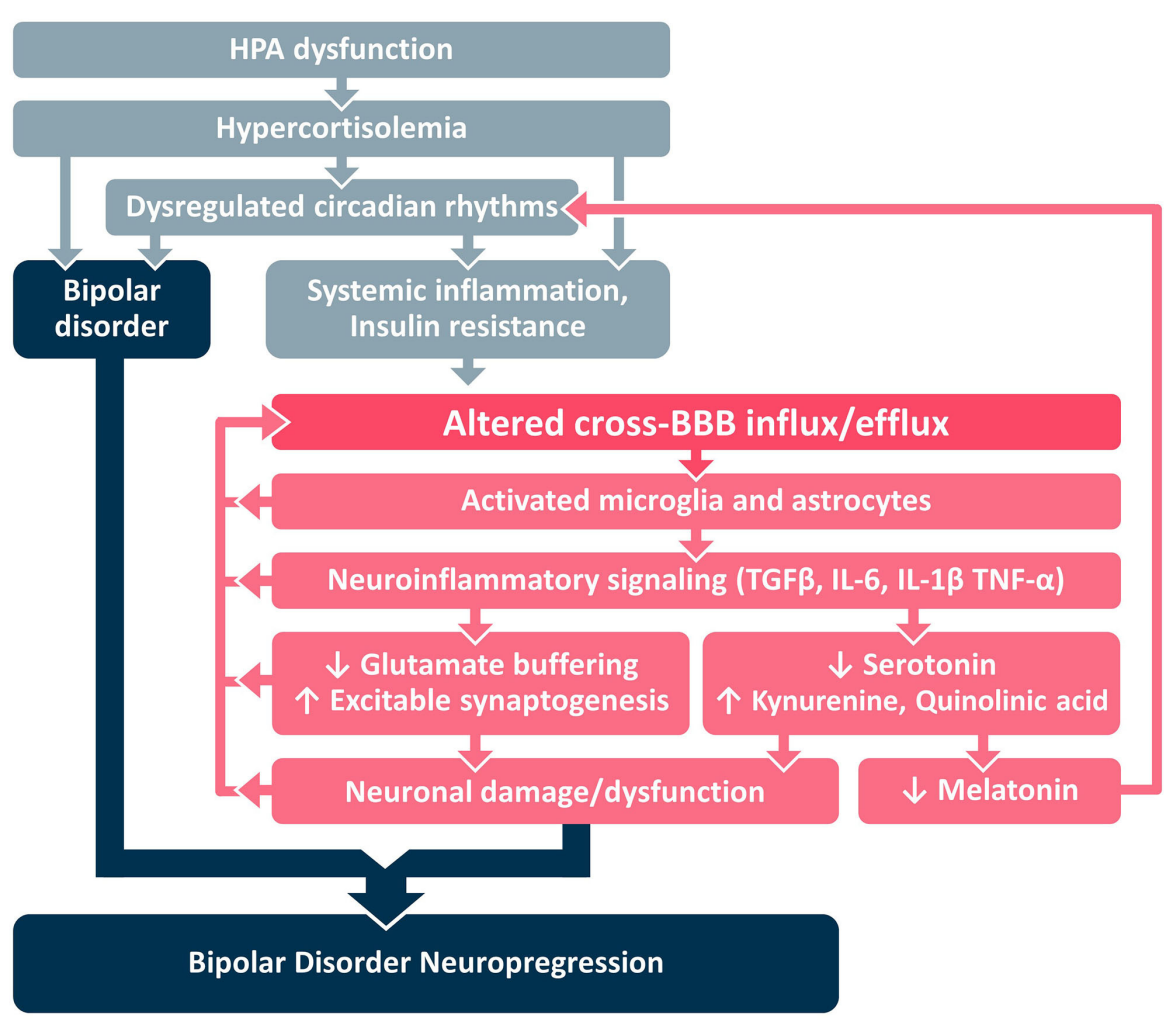
Proposed mechanistic framework. BBB, blood-brain barrier; HPA, hypothalamic pituitary adrenal; IL, interleukin; TGFβ, Transforming Growth Factor Beta; TNF, tumor necrosis factor.

In this paper we present a framework for development of BD neuroprogression (Figure 1B), centered around the transition from systemic inflammation to neuroinflammation via the BBB. We identify HPA dysfunction as an early stage of a pathological cascade that leads to sustained hypercortisolemia and circadian dysregulation that may underlie the parallel development of both (a) BD and (b) systemic inflammation and IR (Figure 2). Systemic inflammation and IR leads to neuroinflammation via cross-BBB infiltration of systemically-secreted cytokines (e.g., IL1, IL-6, or TNF-α) and/or damage to the BBB's endothelium mediated by hyperinsulinemia/hyperglycemia (8, 17, 98, 99). Once the BBB is breached, the brain's microglial and astrocytic cells undergo a neuroinflammatory transformation, involving TGFβ pathway activation and further cytokine secretion. The astrocytic TGFβ cascade results in reduced buffering of extracellular glutamate (100), and generation of new excitatory synapses (91). Together these changes result in a reorganization of the neural network, a shift favoring hyperexcitation, and glutamate-mediated neuronal damage (101). Notably, each step of the neuroinflammatory cascade can contribute to further BBBD, creating a self-reinforcing positive feedback loop that may also amplify the subsequent neuronal dysfunction. We hypothesize that the processes mediated by BBBD and neuroinflammation may impair the function of the affected brain regions, and contribute to the severity of BD and its responsiveness to available mood-stabilizing treatments. Interestingly, neuroinflammatory signaling cascades may also amplify the circadian dysregulation in BD patients, by favoring the production of kynurenine (and quinolinic acid) over serotonin (and melatonin) (102–104). Together, these pathways demonstrate the tight interplay between HPA activity, systemic inflammation, BBBD, neuroinflammation and BD neuroprogression, with the BBB being the interface between systemic and CNS processes/manifestations.

## Can Treating IR And/Or Repairing BBBD Halt BD Neuroprogression?

Thus far, we have reviewed evidence suggesting that: (i) BBBD in bipolar patients might be caused by inflammatory processes such as IR, and (ii) BBBD may contribute to BD neuroprogression, that is more common in bipolar patients with IR. Research into manipulation of the BBB is becoming an area of growing interest, with several groups developing therapeutics targeting the repair of the BBB and/or inhibition of neuroinflammatory processes subsequent to BBBD (105–107). Given that BBBD in bipolar patients may be caused by systemic inflammation and IR, the question follows whether reversal of inflammation or IR could facilitate BBB repair and the remission of neuroprogression.

While some studies suggest that anti-inflammatory treatment or TNF inhibitors may *reduce* depressive symptoms (48, 49), whether they can repair the BBB remains unclear. A study in patients undergoing cardiac surgery suggests that treatment with the potent anti-inflammatory prednisone does not reduce post-operative BBBD (108). While this is perhaps far removed from BD, conceptually, this suggests that treating systemic inflammation alone may not be sufficient to repair the BBB.

Studies in animals suggest that reversal of IR may be a more promising therapeutic target for BBB repair. Specifically, metformin—regarded as the safest and most effective insulin-sensitizing drug—has been reported to reduce BBBD in a mouse model of stroke (109). In a mouse model of systemic inflammation using peripheral injection of bacterial lipopolysaccharide (LPS), metformin was shown to downregulate neuroinflammation and improve exploratory behavior (110). In this model, LPS was shown to cause neuroinflammation (elevated levels of TNF-α and IL-6 in the brain), BBBD and significant lethargy and illness (88, 110). Remarkably, pre-treatment of mice with metformin significantly reduced systemic and CNS inflammation, LPS-induced oxidative stress, and neurobehavioral symptoms of illness (110). In humans, metformin has also been shown to enact antioxidant and anti-inflammatory effects, and to reduce cardiovascular complications, stroke, certain cancers, thyroid diseases, and polycystic ovarian syndrome (111–113).

Metformin is generally understood to exert its effects on the liver and peripheral tissues, by decreasing glucose output from the liver and increasing glucose utilization at peripheral tissues, including the musculature. This process requires the activation of adenosine monophosphate-activated protein kinase (AMPK) which reduces energy expenditure at the cellular level (112). However, metformin may offer further benefits beyond reversing IR. Metformin is also thought to suppresses the action of matrix metalloproteinase-9 (MMP-9)—an enzyme that degrades components of the BBB (114). MMP-9 has been widely implicated in conditions such as cancer, MS, migraines, neuropsychiatric disorders (115, 116), and complications of coronary artery disease (116), atherosclerosis (117), and hypertension (118). Furthermore, increased levels of MMP-9 were demonstrated in bipolar depression (119), suggesting that MMP-9 could contribute to BBB degradation and disease progression of bipolar patients. Metformin has been shown to suppress MMP9 in human breast cancer cell lines, and is gaining focus as a potential anticancer drug (120).

An additional mechanism by which metformin may exert its neuroprotective effect is through activation of the peroxisome proliferator-activated receptor (PPAR) (121). PPAR is thought to mediate the insulin-sensitizing action of metformin, by modulating the insulin-like growth-factor (IGF) axis (121). In animal studies, PPAR agonists (thiazoladinediones, such as rosiglitosone) have been shown to protect the BBB; reduce neuro-inflammation, oxidative stress and neuronal injury (120, 122); and improve neurological outcomes of CNS injury/disease (123). Elegantly, PPAR-γ antagonists lead to an opposite effect (122). These findings were further confirmed in a monolayer of human microvascular endothelial cells (122), suggesting that activation of PPAR signaling may be a promising neuroprotective target, and further highlighting the therapeutic potential of metformin and the thiazolidinedione class of drugs. Targeting the BBB directly, may indeed provide a promising approach in other CNS diseases, such as epilepsy which is also associated with BBBD (77). The angiotensin II receptor antagonist, losartan is vascular protective and has potential implications for protecting BBB integrity though action on TGF-ß. In a rat model of vascular injury, losartan prevented acquired epilepsy via TGF-ß signaling suppression when administered prior to injury and may become the first available treatment for the prevention of epilepsy (105).

We conclude that treatment of IR and potential repair of the BBB with metformin, thiazolidinediones or vascular protective drugs, such as losartan may prove to be effective strategies for treatment resistant or neuroprogressive bipolar disorder (Figure 1D). Further research is needed to investigate the mechanisms underlying the effects of these agents on MMP9 and PPAR pathways in particular. By more directly targeting the BBB, a shift in focus toward *prevention* of BD neuroprogression could emerge.

## Evaluating IR And BBBD To Identify BD Patients With Neuroprogression

Insulin resistance can be easily estimated using concurrent FPG and FSI levels and the HOMA-IR equation: HOMA-IR = FPG (mmol/L) × FSI (μU/ml)/22.5 (124), and the HOMA-IR cut-off value of ≥1.8 (since metabolic syndrome becomes clinically significant at this value) (125). Once it is determined that a patient is insulin resistant, DCE-MRI could be used to quantify and localize BBB leakage. While DCE-MRI is clinically available and there are algorithms to analyze and interpret images for BBBD, this has only been used for research. Standardizing abnormal BBB permeability cut-off values between different types of MRI scanners is required and feasible, but calls for large sample sizes, controls and further validation before it can be brought widely into clinical use. As our group is currently doing this work, we expect BBB imaging to be available for clinical use in the future. We suggest that utilizing these two biomarkers (IR, and BBBD once clinically available) will be important in identifying bipolar patients with neuroprogression and for following treatment response.

## Discussion

We have highlighted the evidence for a key role for IR and shared inflammatory pathways leading to BBBD and neuroprogression of BD. We have outlined the rationale for our expanded hypothesis that these modulations result in BBB leakage and worse outcomes in BD. We have further connected this body of evidence with key known treatments for IR. Indeed, reversing IR could provide a novel strategy for the prevention or treatment of a neuroprogressive course of BD. This could be accomplished through the actions of widely used diabetic medications, such as metformin or thiazolidinediones or vascular protective agents, like losartan, that target BBBD more directly. While there is currently a lack of evidence to clearly determine whether treating IR or BBBD is of value in BD neuroprogression, our group has a number of studies under way. We are hopeful that data from our completed TRIO-BD quadruple-masked randomized clinical trial comparing metformin to placebo in improving outcomes in treatment resistant bipolar depression will soon help provide answers. Further research is needed in terms of replicating completed studies and investigating novel new treatments for BD neuroprogression, targeting IR and BBBD.

## Data Availability Statement

The original contributions presented in the study are included in the article/supplementary material, further inquiries can be directed to the corresponding author/s.

## Author Contributions

CC contributed intellectual concepts, hypotheses, writing, and editing. CM synthesized these concepts and hypotheses in the writing of the manuscript and provided the literature search. LK, KC, and AF contributed to intellectual content, writing, and editing. All authors contributed to the article and approved the submitted version.

## Conflict of Interest

The authors declare that the research was conducted in the absence of any commercial or financial relationships that could be construed as a potential conflict of interest.
